# Supplementary material for: Multi-Scenario Species Distribution Modeling
Source: Insects. 2019 Mar 1;10(3):65. doi: 10.3390/insects10030065 (PMC6468778; doi:10.3390/insects10030065)
Supplement: Supplementary file 1 [file insects-10-00065-s001.pdf]

S1: Canonical correlation analysis results

Table S1.1: Canonical correlation analysis of 48 model component variables (TDaa, TDag, TDdvv, TDtp, TDvv, Pp2, Pp3, DRdr2, DRdr3, MTlogr, MTqda, MTsvm, TDag:Pp2, TDdvv:Pp2, TDtp:Pp2, TDvv:Pp2, TDag:Pp3, TDdvv:Pp3, TDtp:Pp3, TDvv:Pp3, TDag:DRdr2, TDdvv:DRdr2, TDtp:DRdr2, TDvv:DRdr2, TDag:DRdr3, TDdvv:DRdr3, TDtp:DRdr3,TDvv:DRdr3, Pp2:DRdr2, Pp3:DRdr2, Pp2:DRdr3, Pp3:DRdr3, TDag:Pp2:DRdr2, TDdvv:Pp2:DRdr2, TDtp:Pp2:DRdr2, TDvv:Pp2:DRdr2, TDag:Pp3:DRdr2, TDdvv:Pp3:DRdr2, TDtp:Pp3:DRdr2, TDvv:Pp3:DRdr2, TDag:Pp2:DRdr3, TDdvv:Pp2:DRdr3, TDtp:Pp2:DRdr3, TDvv:Pp2:DRdr3, TDag:Pp3:DRdr3, TDdvv:Pp3:DRdr3, TDtp:Pp3:DRdr3, TDvv:Pp3:DRdr3) with 5 performance measure variables (Kappa, AUC, Sensitivity, Specificity, cverror)

	CanR	CanRSQ	F-Value	NumDF	DenDF	Eigen	Wilks Lambda	% var explained	% cum var explained	P. Value	
1	0.9034	**0.8161**	**3.53**	240	638	4.439	**0.01487**	**52.352**	52.35	0	***
2	0.8405	0.7064	2.40	188	513	2.4055	0.08087	28.37	80.72	0	***
3	0.6289	0.3956	1.51	138	388	0.6544	0.27541	7.718	88.44	0.00115	**
4	0.6278	0.3941	1.39	90	260	0.6504	0.45565	7.671	96.11	0.0238	*
5	0.498	0.248	0.98	44	131	0.3298	0.75201	3.889	100	0.51324	ns

#Signif. P codes: 0 < *** ≤ 0.001 < ** ≤ 0.01 < * ≤ 0.05 < . ≤ 0.1 < ns ≤ 1;
Values reported in the manuscript are indicated in bold red figures.

Table S1.2: Canonical covariate loadings (coefficients)

$X.xscores

	Xcan1	Xcan2	Xcan3	Xcan4	Xcan5
TDaa	-0.3157	-0.0725	0.0812	0.0348	0.1076
TDag	-0.0768	0.0676	-0.1318	-0.1375	0.0689
TDdvv	0.1322	0.0393	-0.0392	-0.1626	-0.1598
TDtp	0.6566	-0.0538	0.1230	0.2030	0.0773
TDvv	-0.3963	0.0193	-0.0333	0.0623	-0.0940
Pp2	0.0763	0.0755	0.0651	-0.0148	-0.0546
Pp3	-0.0971	-0.1621	-0.1277	0.0426	0.0354
DRdr2	-0.0720	-0.2462	0.3582	-0.0004	0.0920
DRdr3	-0.0612	0.3430	0.0894	0.0299	0.0526
MTlogr	0.2543	-0.5559	0.0621	0.0241	0.1414
MTqda	0.0833	0.0516	-0.4159	0.0264	0.1427
MTsvm	-0.2426	0.2920	0.0070	-0.0468	0.2329
TDag:Pp2	0.1296	0.0972	-0.1994	-0.2072	-0.0166
TDdvv:Pp2	0.1517	0.0597	0.2131	-0.0494	-0.1897
TDtp:Pp2	0.2383	0.0489	-0.0077	0.2401	0.0851
TDvv:Pp2	-0.1942	0.0019	0.0079	-0.0167	0.0346
TDag:Pp3	-0.1438	-0.1202	-0.2080	0.0449	0.0573
TDdvv:Pp3	0.0938	0.1040	-0.1144	-0.1825	0.0037
TDtp:Pp3	0.3397	-0.3071	0.0652	0.1509	-0.0570
TDvv:Pp3	-0.2493	0.0238	-0.0094	0.0446	-0.0160
TDag:DRdr2	-0.1159	-0.1376	0.0290	-0.0115	0.1702
TDdvv:DRdr2	0.0707	0.0491	0.1459	-0.0879	0.1017
TDtp:DRdr2	0.2691	-0.1916	0.2450	0.1168	-0.1744

$X.yscores

	Ycan1	Ycan2	Ycan3	Ycan4	Ycan5
TDaa	-0.2852	-0.0609	0.0511	0.0219	0.0536
TDag	-0.0694	0.0569	-0.0829	-0.0863	0.0343
TDdvv	0.1195	0.0330	-0.0246	-0.1021	-0.0796
TDtp	0.5931	-0.0452	0.0774	0.1274	0.0385
TDvv	-0.3580	0.0162	-0.0210	0.0391	-0.0468
Pp2	0.0690	0.0635	0.0410	-0.0093	-0.0272
Pp3	-0.0878	-0.1363	-0.0803	0.0267	0.0176
DRdr2	-0.0650	-0.2070	0.2253	-0.0002	0.0458
DRdr3	-0.0553	0.2882	0.0562	0.0187	0.0262
MTlogr	0.2298	-0.4672	0.0391	0.0151	0.0704
MTqda	0.0753	0.0434	-0.2616	0.0166	0.0711
MTsvm	-0.2192	0.2454	0.0044	-0.0294	0.1160
TDag:Pp2	0.1170	0.0817	-0.1254	-0.1301	-0.0083
TDdvv:Pp2	0.1371	0.0502	0.1341	-0.0310	-0.0945
TDtp:Pp2	0.2153	0.0411	-0.0048	0.1507	0.0424
TDvv:Pp2	-0.1755	0.0016	0.0050	-0.0105	0.0172
TDag:Pp3	-0.1299	-0.1010	-0.1308	0.0282	0.0286
TDdvv:Pp3	0.0847	0.0874	-0.0720	-0.1146	0.0018
TDtp:Pp3	0.3069	-0.2581	0.0410	0.0947	-0.0284
TDvv:Pp3	-0.2253	0.0200	-0.0059	0.0280	-0.0080
TDag:DRdr2	-0.1047	-0.1157	0.0182	-0.0072	0.0847
TDdvv:DRdr2	0.0639	0.0413	0.0918	-0.0552	0.0506
TDtp:DRdr2	0.2431	-0.1610	0.1541	0.0733	-0.0868

TDvv:DRdr2	-0.2252	0.0089	0.0757	-0.0203	0.0345
TDag:DRdr3	-0.0001	0.2632	-0.0467	-0.1206	-0.0670
TDdvv:DRdr3	-0.1379	0.0183	-0.0221	0.0368	-0.1409
TDtp:DRdr3	0.4586	0.2389	0.2329	0.0123	0.4257
TDvv:DRdr3	-0.2408	0.0262	0.0077	0.0635	-0.1206
Pp2:DRdr2	0.0653	-0.0904	0.2545	-0.2041	0.0854
Pp3:DRdr2	-0.0118	-0.1225	0.0655	0.1559	-0.0259
Pp2:DRdr3	-0.0177	0.2480	-0.0364	-0.0739	0.0000
Pp3:DRdr3	-0.1888	-0.1139	0.1023	-0.0846	0.1123
TDag:Pp2:DRdr2	0.0929	-0.0831	-0.0566	-0.0525	0.1601
TDdvv:Pp2:DRdr2	0.1515	-0.0047	0.3598	-0.2797	0.0566
TDtp:Pp2:DRdr2	0.0829	0.0458	-0.0689	-0.0546	-0.0533
TDvv:Pp2:DRdr2	-0.1281	0.0038	0.1133	-0.0498	0.0409
TDag:Pp3:DRdr2	-0.1146	-0.0785	-0.0992	-0.0011	-0.0629
TDdvv:Pp3:DRdr2	0.0574	0.1110	-0.0898	0.0336	0.1140
TDtp:Pp3:DRdr2	0.2826	-0.2269	0.2333	0.2851	-0.3160
TDvv:Pp3:DRdr2	-0.1282	-0.0062	0.0147	-0.0068	0.0886
TDag:Pp2:DRdr3	0.1150	0.2553	-0.1282	-0.1753	-0.0586
TDdvv:Pp2:DRdr3	-0.0551	0.0771	0.0086	-0.1326	-0.2414
TDtp:Pp2:DRdr3	0.1746	0.1047	0.0285	0.1464	0.3407
TDvv:Pp2:DRdr3	-0.1434	0.0590	0.0170	0.0047	-0.0211
TDag:Pp3:DRdr3	-0.1564	-0.0723	0.0340	-0.0376	0.1554
TDdvv:Pp3:DRdr3	-0.0891	-0.0231	-0.0230	0.0974	0.0015
TDtp:Pp3:DRdr3	0.1494	-0.2105	0.1568	-0.2800	0.1379
TDvv:Pp3:DRdr3	-0.1584	0.0324	0.0184	0.0272	-0.0421

$Y.xscores

	Xcan1	Xcan2	Xcan3	Xcan4	Xcan5
kappa	**-0.7219**	0.41643	-0.06919823	0.20104145	0.0179
auc	-0.5967	0.48672	-0.21969584	0.06150155	0.15494
Sensitivity	-0.7179	0.33381	0.1467134	0.19945241	0.11726
Specificity	-0.5754	0.48431	-0.2355396	0.20181835	-0.0681
cverror	0.68693	**-0.5269**	0.05577964	-0.08332753	-0.0286

TDvv:DRdr2	-0.2034	0.0075	0.0476	-0.0127	0.0172
TDag:DRdr3	-0.0001	0.2212	-0.0294	-0.0757	-0.0334
TDdvv:DRdr3	-0.1246	0.0153	-0.0139	0.0231	-0.0702
TDtp:DRdr3	0.4143	0.2007	0.1465	0.0077	0.2120
TDvv:DRdr3	-0.2175	0.0220	0.0049	0.0398	-0.0601
Pp2:DRdr2	0.0590	-0.0759	0.1600	-0.1281	0.0425
Pp3:DRdr2	-0.0107	-0.1030	0.0412	0.0979	-0.0129
Pp2:DRdr3	-0.0160	0.2084	-0.0229	-0.0464	0.0000
Pp3:DRdr3	-0.1706	-0.0957	0.0643	-0.0531	0.0559
TDag:Pp2:DRdr2	0.0839	-0.0698	-0.0356	-0.0330	0.0797
TDdvv:Pp2:DRdr2	0.1369	-0.0040	0.2263	-0.1756	0.0282
TDtp:Pp2:DRdr2	0.0749	0.0385	-0.0433	-0.0343	-0.0266
TDvv:Pp2:DRdr2	-0.1158	0.0032	0.0712	-0.0313	0.0204
TDag:Pp3:DRdr2	-0.1036	-0.0659	-0.0624	-0.0007	-0.0313
TDdvv:Pp3:DRdr2	0.0518	0.0933	-0.0565	0.0211	0.0568
TDtp:Pp3:DRdr2	0.2553	-0.1907	0.1468	0.1789	-0.1573
TDvv:Pp3:DRdr2	-0.1158	-0.0052	0.0092	-0.0043	0.0441
TDag:Pp2:DRdr3	0.1039	0.2146	-0.0806	-0.1101	-0.0292
TDdvv:Pp2:DRdr3	-0.0497	0.0648	0.0054	-0.0832	-0.1202
TDtp:Pp2:DRdr3	0.1577	0.0880	0.0179	0.0919	0.1697
TDvv:Pp2:DRdr3	-0.1296	0.0496	0.0107	0.0029	-0.0105
TDag:Pp3:DRdr3	-0.1413	-0.0608	0.0214	-0.0236	0.0774
TDdvv:Pp3:DRdr3	-0.0805	-0.0194	-0.0145	0.0612	0.0007
TDtp:Pp3:DRdr3	0.1350	-0.1769	0.0986	-0.1757	0.0687
TDvv:Pp3:DRdr3	-0.1431	0.0272	0.0116	0.0171	-0.0210

$Y.yscores

	Ycan1	Ycan2	Ycan3	Ycan4	Ycan5
kappa	**-0.7990843**	0.4954877	-0.11	0.32025	0.03595
auc	-0.6604577	0.5791152	-0.3493	0.09797	0.31113
Sensitivity	-0.7946309	0.3971826	0.23327	0.31772	0.23548
Specificity	-0.6369051	0.5762463	-0.3745	0.32148	-0.1367
cverror	0.7603776	**-0.6269267**	0.08869	-0.1327	-0.0575

Values reported in the manuscript are indicated in bold red figures.

Keys for abbreviations used in S1.1 and S1.2

- TDaa: Species training dataset *Aedes albopictus*; TDag: Species training dataset *Anoplopis gracilipes;* TDdvv: Species training dataset *Diabrotica v. virgifera;* TDtp: Species training dataset *Thaumetopoea pityocampa*; TDvv: Species training dataset *Vespula vulgaris.*
- Pp1: Predictor dataset BIOCLIM19 (P1); Pp2: Predictor dataset BIOCLIM35 (P2); Pp3: Predictor dataset BIOCLIM35+T4 (P3).
- DRdr1: Dimension-reduction method RF; DRdr2: Dimension-reduction method PCA; DRdr3: Dimension-reduction method h-NLPCA.
- MTqda: Model type QDA; MTlogr: Model type LOGR; MTcart: Model type CART; MTsvm: Model type SVM.

Table S1.3: Correlation between the canonical scores of canonical dimension X (model components) and canonical dimension Y (model performance measures).

	Ycan1	Ycan2	Ycan3	Ycan4	Ycan5
**Xcan1**	0.90341	0	0	0	0
**Xcan2**	0	0.84045	0	0	0
**Xcan3**	0	0	0.6289378	0	0
**Xcan4**	0	0	0	0.6277696	0
**Xcan5**	0	0	0	0	0.49799

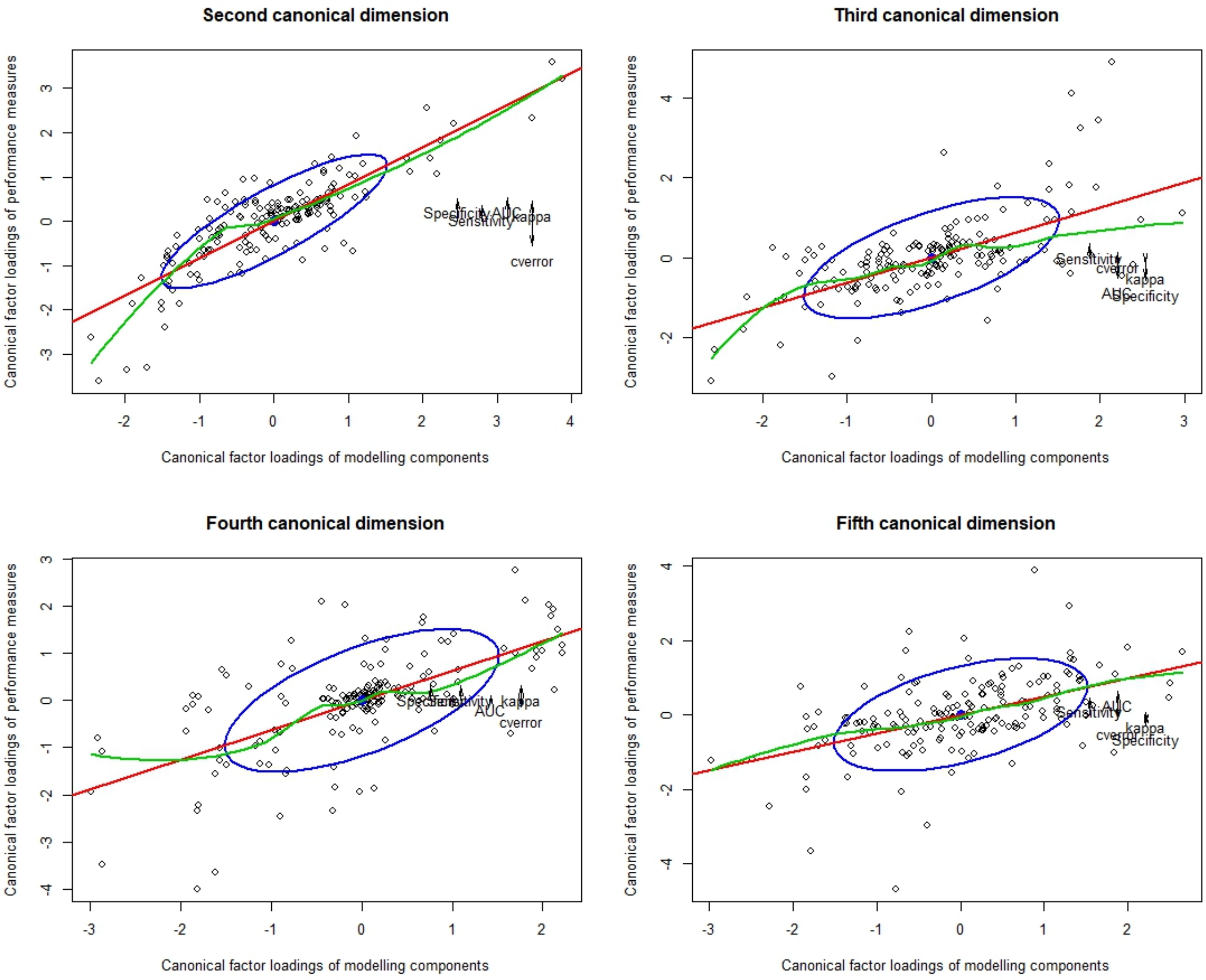

Figure S1.4 The canonical factor loadings for the 2nd -5th dimension. (Subsequent graphs to the 1st dimension graph given in Figure 4 of the manuscript).

Structure correlations (canonical factor loadings) for the second – fifth canonical dimensions. Arrows show the vector direction of variables that correspond to the canonical component on the y-axis. The corresponding variables for the x-axis (combinations of modelling components) were not labelled so not to overcrowd the graph. Red line indicates the linear regression line, blue ellipse (data ellipse) shows 68% of the data points (approx. 1SD) and their centroid (filled black dot) in relation to the linear regression line, green line shows the locally weighted scatterplot smoothing (LOWESS) fit.

Table S2: Ranks of variables based on proportion of inclusion in models

Rank	Variables	Proportion	Dataset	Order number in Table 1
1	Annual precipitation (mm)	0.67	P1, P2, P3	12
1	Precipitation of wettest quarter (mm)	0.67	P1, P2, P3	16
2	Precipitation of driest quarter (mm)	0.44	P1, P2, P3	17
3	Precipitation of warmest quarter (mm)	0.40	P1, P2, P3	18
4	Mean temperature of wettest quarter (°C)	0.38	P1, P2, P3	8
4	Precipitation of coldest quarter (mm)	0.38	P1, P2, P3	19
5	Annual mean temperature (°C)	0.36	P1, P2, P3	1
6	Mean diurnal temperature range (mean(period max-min)) (°C)	0.33	P1, P2, P3	2
6	Max temperature of warmest week (°C)	0.33	P1, P2, P3	5
7	Lowest weekly radiation (W m-2	0.31	P2, P3	22
8	Min temperature of coldest week (°C)	0.29	P1, P2, P3	6
8	Mean temperature of warmest quarter (°C)	0.29	P1, P2, P3	10
8	Mean temperature of coldest quarter (°C)	0.29	P1, P2, P3	11
8	Radiation of warmest quarter (W m-2)	0.29	P2, P3	26
9	Temperature seasonality (C of V)	0.27	P1, P2, P3	4
10	Mean temperature of driest quarter (°C)	0.24	P1, P2, P3	9
10	Precipitation of driest week (mm)	0.24	P1, P2, P3	14
10	Precipitation seasonality (C of V)	0.24	P1, P2, P3	15
10	Elevation (m)	0.24	P3	36
11	Precipitation of wettest week (mm)	0.22	P1, P2, P3	13
12	Temperature annual range (Bio05-Bio06) (°C)	0.16	P1, P2, P3	7
12	Highest weekly radiation (W m-2)	0.16	P2, P3	21
12	Radiation of driest quarter (W m-2)	0.16	P2, P3	25
13	Annual mean radiation (W m-2)	0.11	P2, P3	20
13	Highest weekly moisture index	0.11	P2, P3	29
14	Radiation seasonality (C of V)	0.09	P2, P3	23
14	Radiation of coldest quarter (W m-2)	0.09	P2, P3	27
14	Mean moisture index of driest quarter	0.09	P2, P3	33
15	Annual mean moisture index	0.07	P2, P3	28
15	Lowest weekly moisture index	0.07	P2, P3	30
15	Moisture index seasonality (C of V)	0.07	P2, P3	31
15	Hillshade	0.07	P3	39
16	Isothermality (Bio02 ÷ Bio07)	0.04	P1, P2, P3	3
16	Mean moisture index of coldest quarter	0.04	P2, P3	35
17	Mean moisture index of wettest quarter	0.02	P2, P3	32
17	Slope (deg)	0.02	P3	37
18	Radiation of wettest quarter (W m-2)	0.00	P2, P3	24
18	Mean moisture index of warmest quarter	0.00	P2, P3	34
18	Aspect (deg)	0.00	P3	38

The grey shaded rows cover variables that lie in the top half (1-9) of the total 18 ranks.

To account for variables that contributed to PCA components the eigenvectors that correspond to principal component scores that explained up to 90% of the variance in the dataset were used (specifically variables with absolute loadings >= 0.32) (Dormann et al., 2013 - & references therein). In the case of NLPCA, due to the non-linear nature of feature extraction, it is not possible to get a single corresponding variable coefficient for the scores, however the final weight matrix was used as a proxy for estimating the major contributing variables toward the high variance principal component scores.

Reference

Dormann, C.F., Elith, J., Bacher, S., Buchmann, C., Carl, G., Carré, G., Marquéz, J.R.G., Gruber, B., Lafourcade, B. & Leitão, P.J. (2013) Collinearity: a review of methods to deal with it and a simulation study evaluating their performance. *Ecography*, **36**, 027-046.

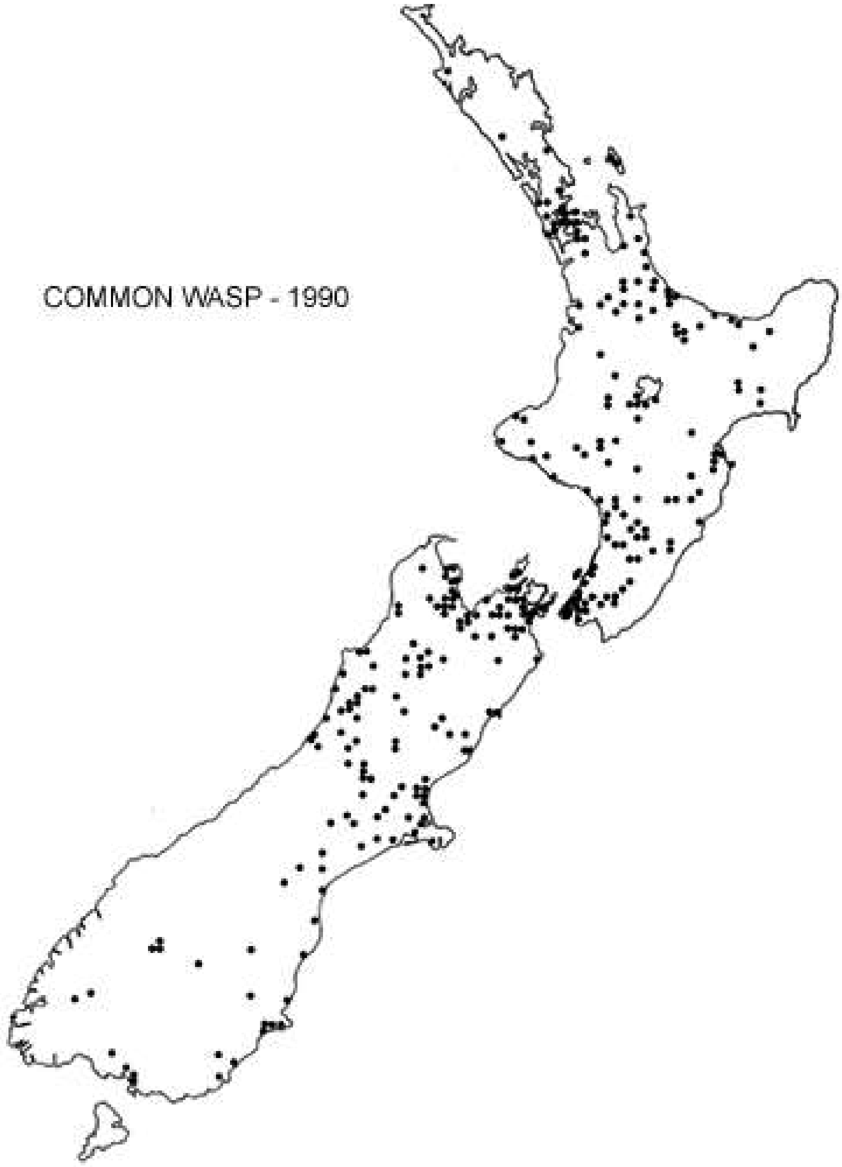

Figure S3: External validation data for *V. vulgaris* in New Zealand

Geographic locations of V. vulgaris presences in New Zealand. Source: AgResearch, Crown Research Institute, New Zealand.

Table S4: Comparison between different modelling component combinations

Table S4.1: TD-DR-MT combinations

No.	Combination	Kappa means	
1	aa.dr3.svm	0.986	a
2	vv.dr3.svm	0.981	a
3	aa.dr2.svm	0.980	ab
4	aa.dr3.cart	0.977	ab
5	vv.dr3.cart	0.969	abc
6	vv.dr2.svm	0.964	abc
7	dvv.dr3.svm	0.954	abc
8	aa.dr2.cart	0.947	abc
9	dvv.dr2.svm	0.943	abc
10	aa.dr1.svm	0.928	abc
11	vv.dr1.svm	0.924	abc
12	vv.dr2.cart	0.917	abc
13	ag.dr3.svm	0.914	abc
14	vv.dr3.qda	0.914	abc
15	aa.dr1.cart	0.903	abc
16	aa.dr3.qda	0.900	abc
17	dvv.dr3.cart	0.887	abcd
18	vv.dr2.qda	0.880	abcd
19	aa.dr1.qda	0.876	abcd
20	vv.dr1.cart	0.871	abcd
21	vv.dr1.qda	0.867	abcd
22	ag.dr3.qda	0.837	abcd
23	ag.dr2.svm	0.835	abcd
24	aa.dr2.qda	0.830	abcd
25	ag.dr3.cart	0.827	abcd

No.	Combination	Kappa means	
26	vv.dr1.log	0.825	abcd
27	ag.dr2.qda	0.808	abcde
28	dvv.dr3.qda	0.789	abcdef
29	ag.dr1.svm	0.788	abcdef
30	aa.dr3.log	0.788	abcdef
31	dvv.dr2.cart	0.784	abcdef
32	vv.dr3.log	0.782	abcdef
33	dvv.dr3.log	0.779	abcdef
34	vv.dr2.log	0.777	abcdef
35	ag.dr2.cart	0.777	abcdef
36	ag.dr1.log	0.772	abcdef
37	ag.dr1.qda	0.765	abcdef
38	tp.dr3.svm	0.764	abcdef
39	ag.dr3.log	0.755	abcdef
40	ag.dr1.cart	0.736	abcdef
41	ag.dr2.log	0.733	abcdef
42	aa.dr1.log	0.723	abcdef
43	tp.dr1.svm	0.699	abcdef
44	dvv.dr1.svm	0.683	abcdef
45	tp.dr3.cart	0.674	abcdef
46	tp.dr2.svm	0.673	abcdef
47	tp.dr2.cart	0.665	abcdef
48	dvv.dr2.qda	0.663	abcdef
49	tp.dr1.qda	0.652	abcdef
50	dvv.dr1.cart	0.622	abcdef

No.	Combination	Kappa means	
51	tp.dr1.cart	0.613	abcdef
52	tp.dr3.qda	0.600	abcdef
53	tp.dr2.qda	0.589	abcdef
54	dvv.dr1.qda	0.569	abcdef
55	dvv.dr2.log	0.526	bcdef
56	dvv.dr1.log	0.517	cdef
57	tp.dr1.log	0.436	def
58	tp.dr2.log	0.367	ef
59	tp.dr3.log	0.350	f
60	aa.dr2.log	0.338	f

Variation in model mean Kappa scores according to different species data (SP), dimension reduction methods (DR) and model types (MT) combinations. Rows with different letters are significantly different (Tukey's HSD test, HSD = 0.45, α = 0.05).

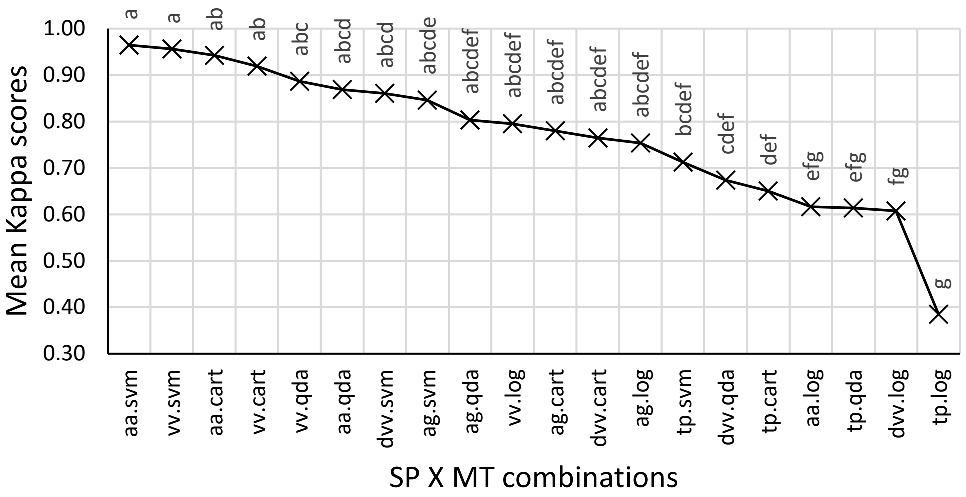

Figure S4.2: Mean Kappa scores for TD-MT combinations

Variation in model mean Kappa scores according to different species data (SP) and model type (MT) combinations. Data points with different letters are significantly different (Tukey's HSD test, HSD = 0.24, α = 0.05)

Keys for the abbreviations of the different TD-DR-MT combinations given in Table S4.1 and Figure S4.2

- TD (Species training data) is represented by the five species modelled: aa for *Aedes albopictus*; ag *Anoplopis gracilipes*; dvv *Diabrotica v. virgifera*; tp for *Thaumetopoea pityocampa*; vv for *Vespula vulgaris.*
- DR (dimension-reduction method) is represented by the 3 dimension-reduction methods used in this study: dr1: RF; dr2: PCA; dr3: h-NLPCA.
- MT (Model type) is represented by the four models used in this study: qda: quantitative discriminant analysis (QDA); logr: logistic regression (LOGR); cart: classification and regression trees (CART); svm: Support Vector Machine (SVM).

Note S5: Distribution predictions for the five species in this study

Species level results were briefly discussed in the results section, here observations related with each species and their associated prediction from the optimum model (the model that had the highest Kappa as well as the lowest cross-validation error) is discussed.

1. *Aedes albopictus* (Skuse, 1894) (Diptera: Culicidae)

The data-dimension reduction-model combination (Fig. 6C) that comprised the optimum model for *A. albopictus* was BIOCLIM19 with the RF variable selection method and SVM model. Areas identified as having a high climatic suitability for *A. albopictus* could further be assessed by using high resolution data along with trade and cargo network information for the target area because used tyres and plant material imports are identified to be the most important introduction pathways for this species (Scholte & Schaffner, 2007; Scholte *et al.*, 2008).

2. *Anoplopis gracilipes* (Smith, 1857) (Hymenoptera: Formicidae)

For *A. gracilipes*, areas of high probability of predicted presence obtained from the selected P_1DR_2QDA model was further assessed by examining the uncertainty map for *A. gracilipes*. The following locations were indicated as highly climatically suitable with overall low uncertainty: Bahi and Amazonas regions of Brazil, the northern coast of Venezuela, Honduras, Nicaragua, coastal areas of Equatorial Guinea, Liberia, Ghana, the western coast of Namibia, south-eastern coast of South Africa (Wetterer, 2005), northern Australia (Hoffmann, 2014) and most islands in the Caribbean and Indian Ocean. *A. gracilipes* is reported to be established in some of the identified areas, even though they were not included in the training or test data as there was no geo-referenced data with the reports. Locations listed with citations are predictions where *A. gracilipes* is already in the country. For New Zealand no high probability areas were predicted. However there was a great deal of variation between *A. gracilipes* distribution predictions for New Zealand by the other models with significantly high Kappa, so maybe this result should be interpreted with caution. On the other hand, *A. gracilipes* have been detected in New Zealand in 2002 in the Auckland area but was later eradicated (Wetterer, 2005). In light of the distribution prediction for *A. gracilipes* in this chapter, it is probable that the success of the eradication could have been enhanced by the unsuitability of the climate in New Zealand.

3. *Diabrotica virgifera virgifera* (LeConte, 1868) (Coleoptera: Chrysomelidae, Galerucinae)

Although the best model for *D. v. virgifera* prediction covered most of the known range of the species, it did not predict the original native range of *D. v. virgifera*, Central America despite presence points from the area being explicitly included in model training. The optimum model combination P_1DR_2SVM predicted the original native range while the model that used RF instead of PCA did not predict the Central American native range. This result implies that models can miss important locations if the appropriate data pre-processing method is not used. Cases where known locations are not predicted even if an occurrence record from the same locality was included in the model training is particularly worrying because modellers will probably not investigate further. Therefore, further study is needed to investigate

such scenarios. Modelling the climatically suitable areas along with maize plantation cover is recommended to prioritize suitable areas at the risk of *D. v. virgifera* invasion (Aragón *et al.*, 2010).

4. *Thaumetopoea pityocampa* (Denis & Schiffermuller, 1775) (Lepidoptera: Thaumetopoeidae)
The selected model P_2DR_2SVM predicted the known geographical ranges of *T. pityocampa* in Europe and Central Asia, except for its distribution in the North Africa (Rousselet *et al.*, 2010). The under prediction was a result of incomplete occurrence data as all the presence points available were from the Mediterranean range of *T. pityocampa* distribution. For New Zealand, most areas in the Manawatu-Wanganui Region, eastern coasts of Canterbury and Otago regions were predicted to be highly climatically suitable for *T. pityocampa* establishment. Because the occurrence data used in this study only covers part of the known ranges of *T. pityocampa*, only positive predictions for the potential distribution of the species were considered. This is essentially because predicted low probability areas might not necessarily show actual unsuitability due to the missed opportunity of matching areas similar to the North African range for which there were no presence points available to this study. In such cases it is advisable to further consult mechanistic model outputs if physiological information is available for the species (Robinet *et al.*, 2007).

5. *Vespula vulgaris* (Linnaeus, 1758) (Hymenoptera: Vespidae)
The SVM model selected for *V. vulgaris* prediction was based on BIOCLIM19 data and a random forest variable selection method. The prediction covered the native Holarctic range of *V. vulgaris* and its introduced range in New Zealand including Stewart Island and Tasmania in Australia (Thomas *et al.*, 1990; Matthews *et al.*, 2000). An external validation carried out for New Zealand using *V. vulgaris* presence data obtained from the website[1] of Landcare Research showed that 91% of the occurrence sites were correctly predicted by the selected model (Appendix S10). Another area identified as a highly suitable was Southern Argentina, *V. vulgaris* was reported from this location in 2010 by Masciocchi *et al.* (2010) but no follow up report on its establishment could be found. However, since the German wasp (*Vespula germanica*) which co-occurs with *V. vulgaris* in New Zealand is present in Argentina (D'Adamo *et al.*, 2002; Lopez-Osorio *et al.*, 2014), it is entirely possible that the climate in the predicted areas of Argentina is also suitable for *V. vulgaris*. If this is the case displacement of *V. germanica* from Argentina is also a possibility according to the trend reported in New Zealand (Harris, 1991). A suitable area of notable size is also predicted in Canada and the U.S.A.

References

Aragón, P., Baselga, A. & Lobo, J.M. (2010) Global estimation of invasion risk zones for the western corn rootworm *Diabrotica virgifera virgifera*: integrating distribution models and physiological thresholds to assess climatic favourability. *Journal of Applied Ecology*, **47**, 1026-1035.

[1]http://www.landcareresearch.co.nz/science/plants-animals-fungi/animals/invertebrates/invasive-invertebrates/wasps/distribution/common

D'Adamo, P., Sackmann, P., Corley, J.C. & Rabinovich, M. (2002) The potential distribution of German wasps (*Vespula germanica*) in Argentina. *New Zealand Journal of Zoology*, **29**, 79-85.

Harris, R.J. (1991) Diet of the wasps *Vespula vulgaris* and *V. germanica* in honeydew beech forest of the South Island, New Zealand. *New Zealand Journal of Zoology*, **18**, 159-169.

Hoffmann, B.D. (2014) Quantification of supercolonial traits in the yellow crazy ant, *Anoplolepis gracilipes*. *Journal of Insect Science (Madison)*, **14**

Lopez-Osorio, F., Pickett, K.M., Carpenter, J.M., Ballif, B.A. & Agnarsson, I. (2014) Phylogenetic relationships of yellowjackets inferred from nine loci (Hymenoptera: *Vespidae, Vespinae, Vespula* and *Dolichovespula*). *Molecular Phylogenetics and Evolution*, **73**, 190-201.

Masciocchi, M., Beggs, J.R., Carpenter, J.M. & Corley, J.C. (2010) Primer registro de *Vespula vulgaris* (Hymenoptera: Vespidae) en la Argentina. *Revista de la Sociedad Entomológica Argentina*, **69**, 267-270.

Matthews, R.W., Goodisman, M.A., Austin, A.D. & Bashford, R. (2000) The introduced English wasp *Vespula vulgaris* (L.)(Hymenoptera: Vespidae) newly recorded invading native forests in Tasmania. *Australian Journal of Entomology*, **39**, 177-179.

Robinet, C., Baier, P., Pennerstorfer, J., Schopf, A. & Roques, A. (2007) Modelling the effects of climate change on the potential feeding activity of *Thaumetopoea pityocampa* (Den. & Schiff.) (Lep., Notodontidae) in France. *Global Ecology and Biogeography*, **16**, 460-471.

Rousselet, J., Zhao, R., Argal, D., Simonato, M., Battisti, A., Roques, A. & Kerdelhué, C. (2010) The role of topography in structuring the demographic history of the pine processionary moth, *Thaumetopoea pityocampa* (Lepidoptera: Notodontidae). *Journal of biogeography*, **37**, 1478-1490.

Scholte, E.J. & Schaffner, F. (2007) 14. Waiting for the tiger: establishment and spread of the *Aedes albopictus* mosquito in Europe. *Emerging pests and vector-borne diseases in Europe*, **1**, 241.

Scholte, E.J., Dijkstra, E., Blok, H., De Vries, A., Takken, W., Hofhuis, A., Koopmans, M., De Boer, A. & Reusken, C.B.E.M. (2008) Accidental importation of the mosquito *Aedes albopictus* into the Netherlands: a survey of mosquito distribution and the presence of dengue virus. *Medical and Veterinary Entomology*, **22**, 352-358.

Thomas, C., Moller, H., Plunkett, G. & Harris, R. (1990) The prevalence of introduced *Vespula vulgaris* wasps in a New Zealand beech forest community. *New Zealand journal of ecology*, **13**, 63-72.

Wetterer, J.K. (2005) Worldwide Distribution and Potential Spread of the Long-Legged Ant, *Anoplolepis gracilipes* (Hymenoptera: Formicidae). *Sociobiology*, **45**, 77-97.

S6: Species distribution maps according to three different modelling component combinations.

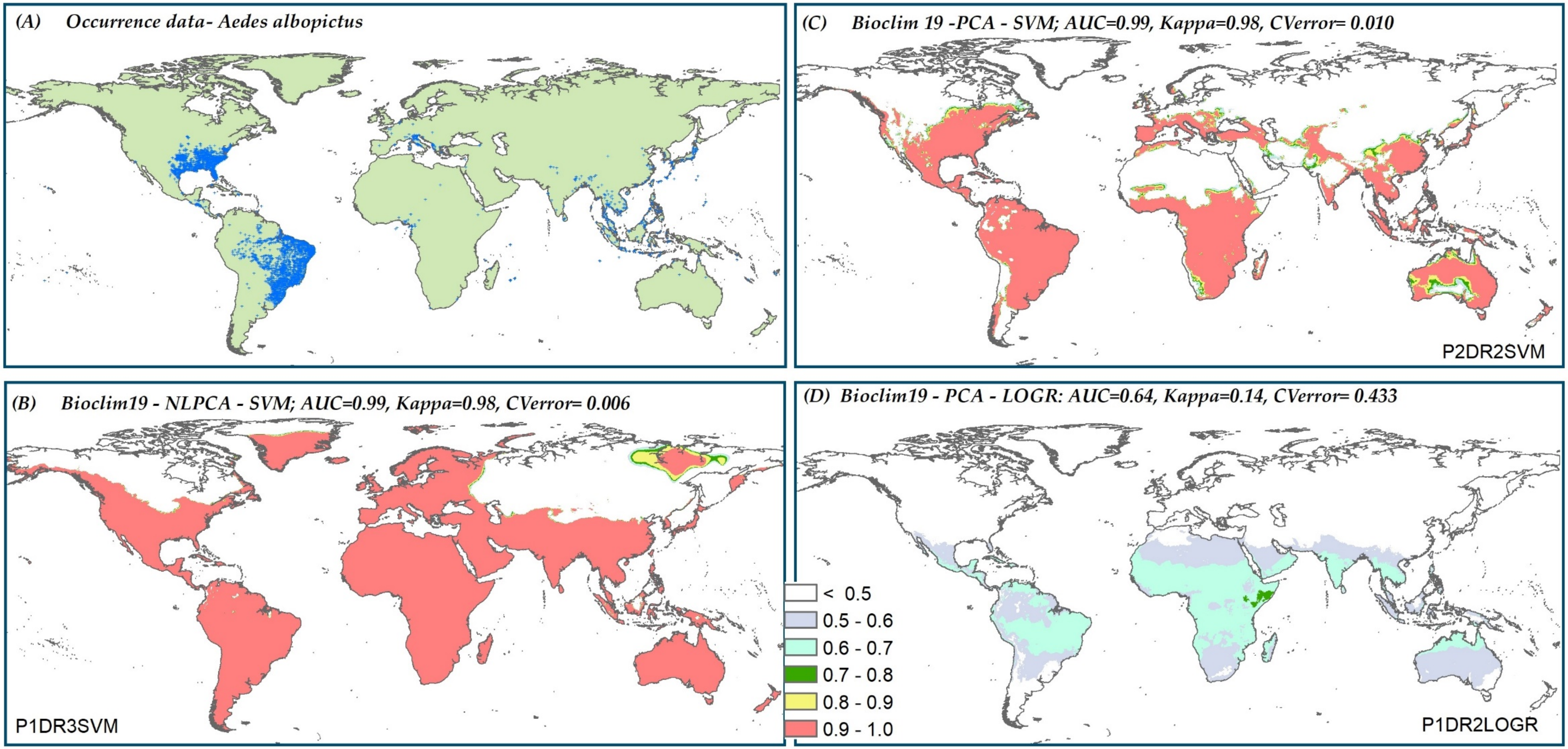

Figure S6.1: Global predicted probability of occurrence for *A. albopictus*: (A) occurrence data, (B) prediction based on the highest Kappa model, (C) Prediction based on the same Kappa score as panel B but with better prediction as per Note S5, (D) Worst Kappa model prediction

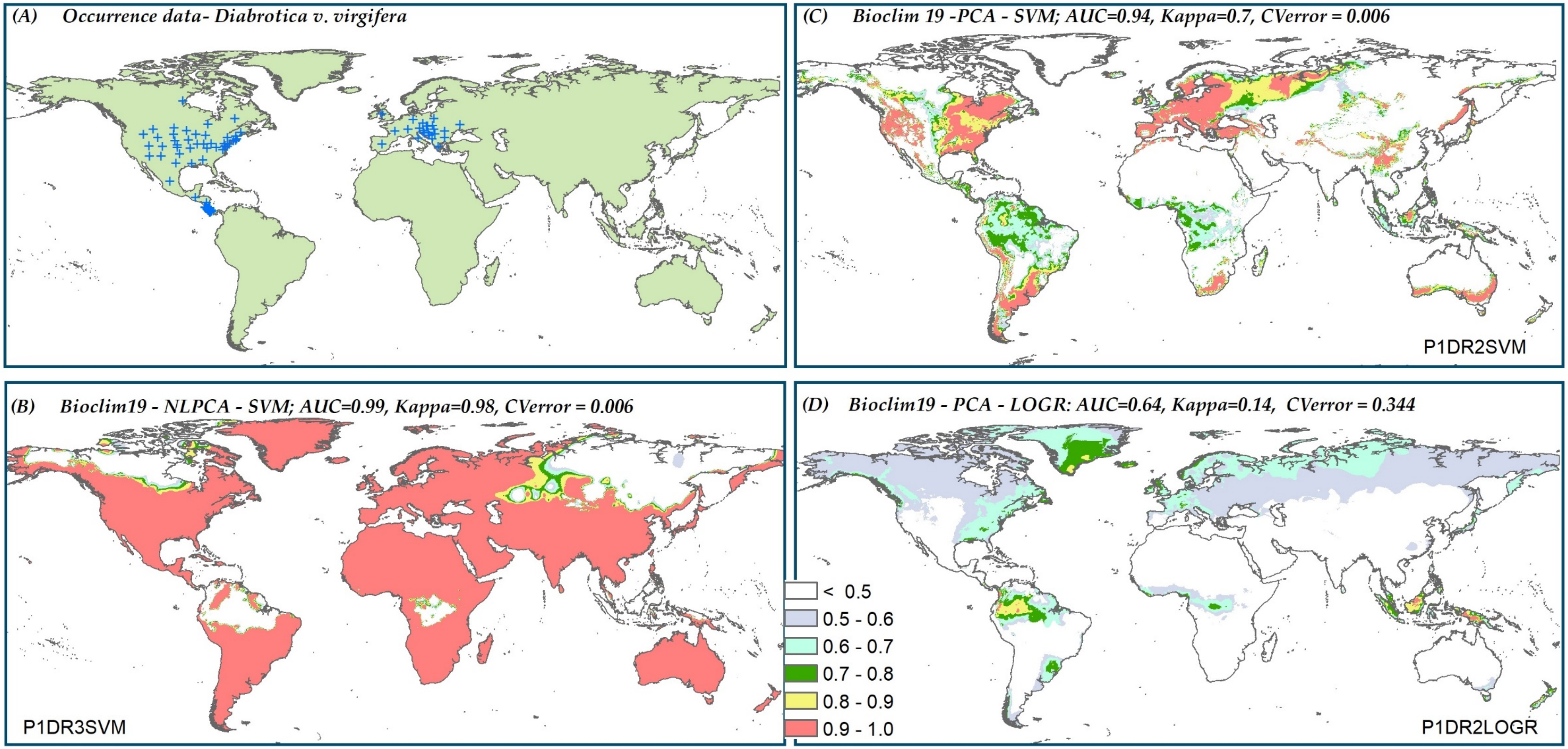

Figure S6.2: Global predicted probability of occurrence for *D. v. virgifera*: (A) occurrence data, (B) prediction based on the highest Kappa score model, (C) prediction based on the second highest Kappa score model but with better prediction as per Note S5, (D) Worst Kappa score model prediction

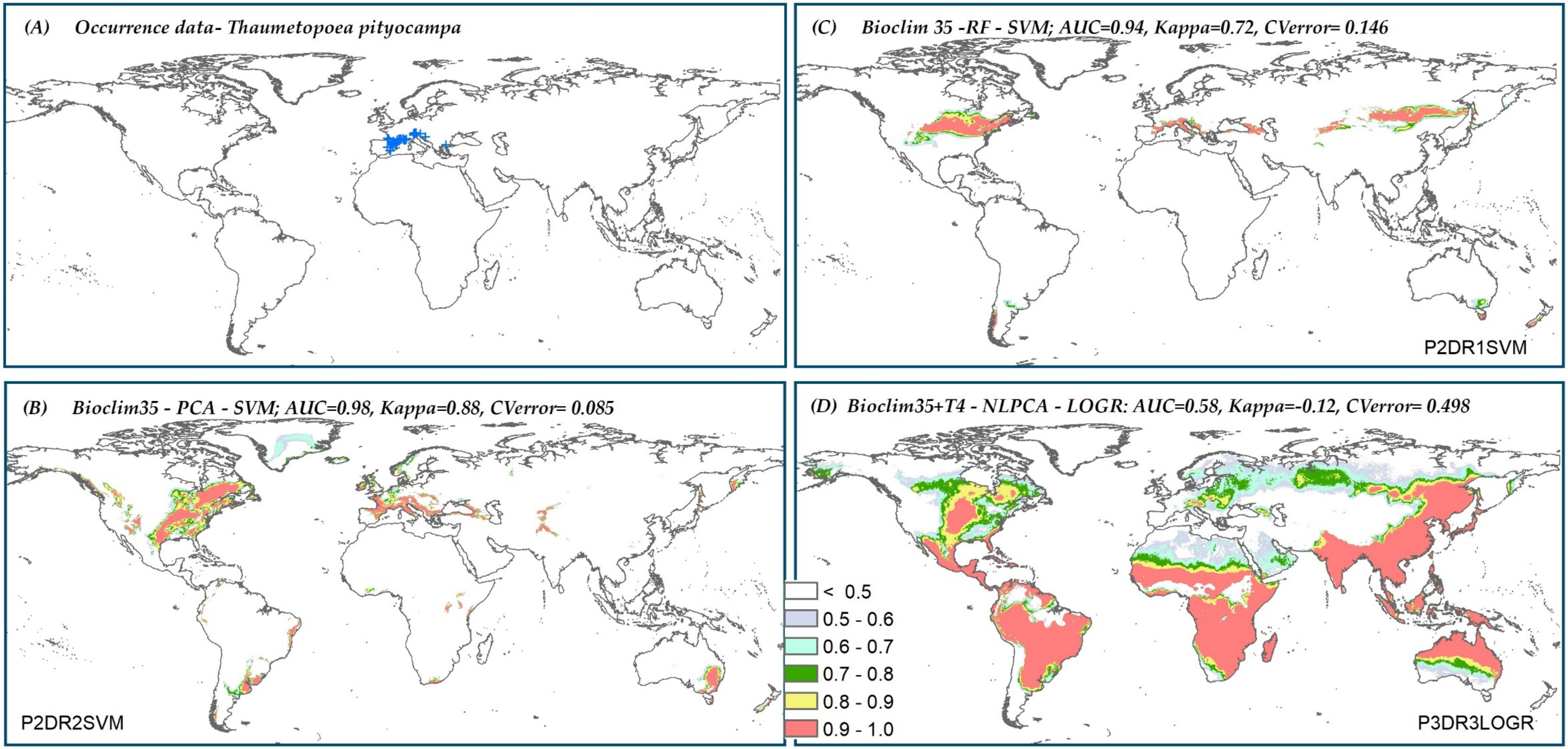

Figure 6.3: Global predicted probability of occurrence for *T. pityocampa*: (A) occurrence data, (B) prediction based on the highest Kappa score model, (C) Prediction based on the second highest Kappa score model, (D) Worst Kappa score model prediction

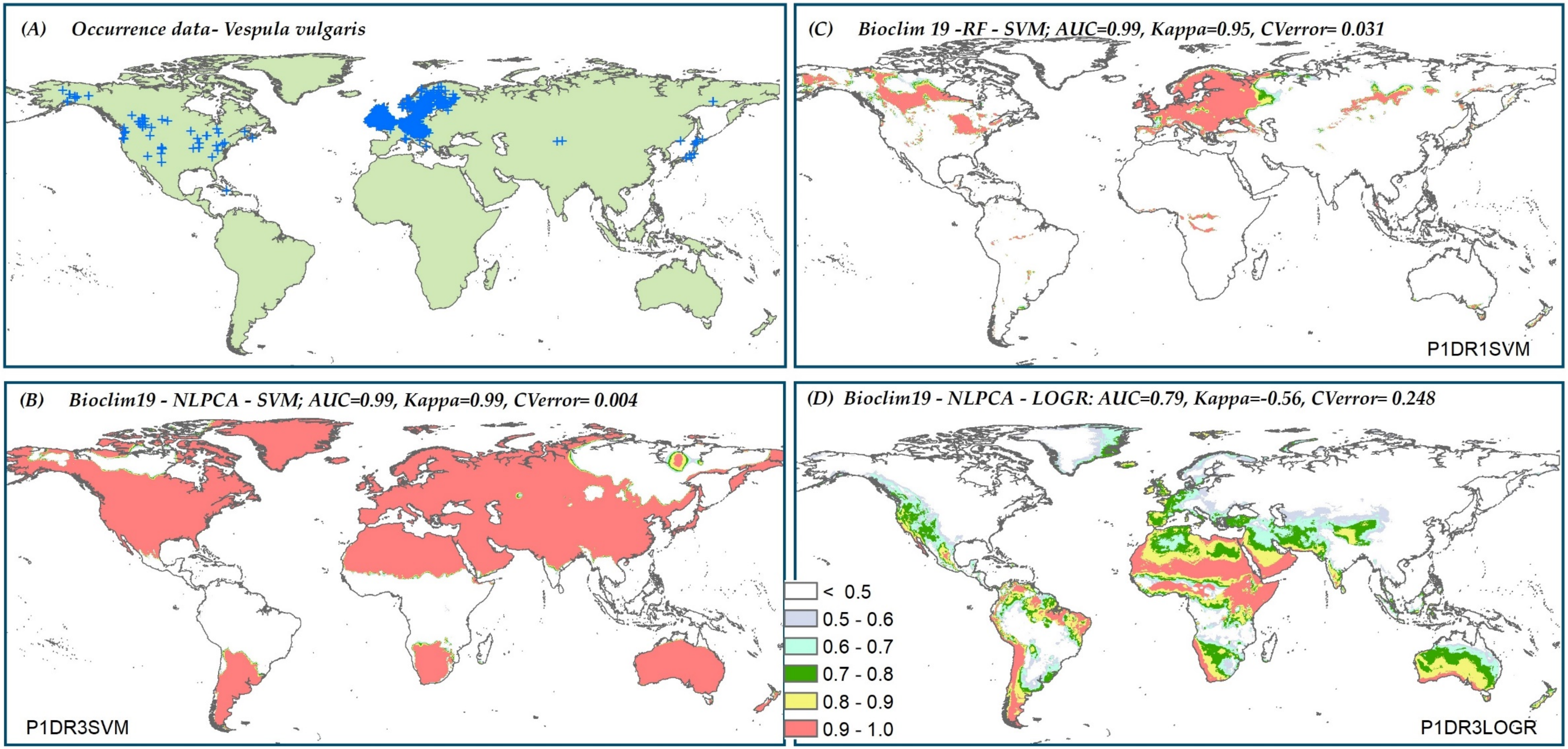

Figure S6.4: Global predicted probability of occurrence for *V. vulgaris*: (A) occurrence data, (B) prediction based on the highest Kappa score model, (C) prediction based on the second highest Kappa score model but with better prediction as per Note S5, (D) Worst Kappa score model prediction

S7: Ensemble mean predictions and uncertainty maps

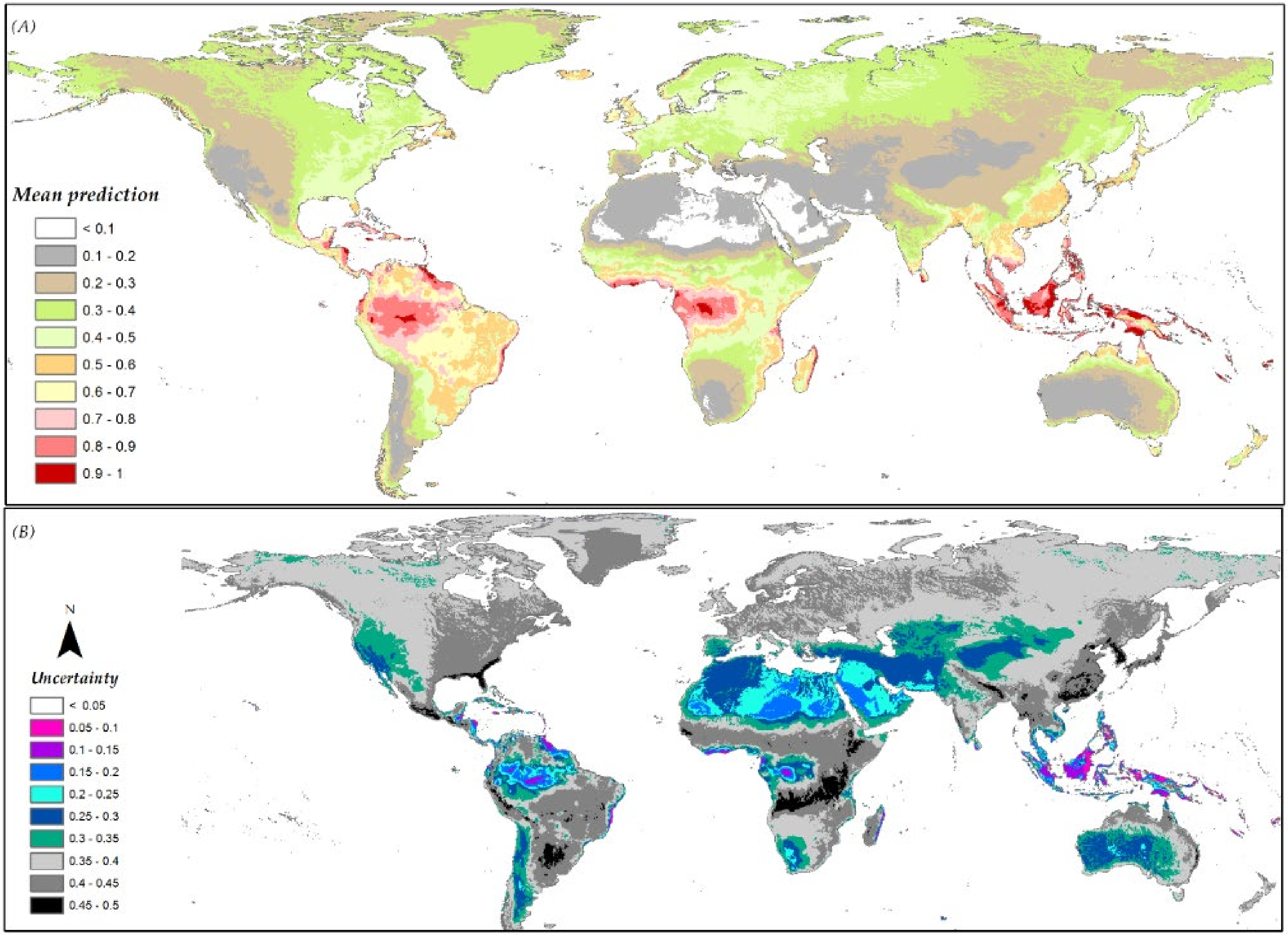

Figure S7.1: Global average prediction (A) and uncertainty map (B) for *A. gracilipes*

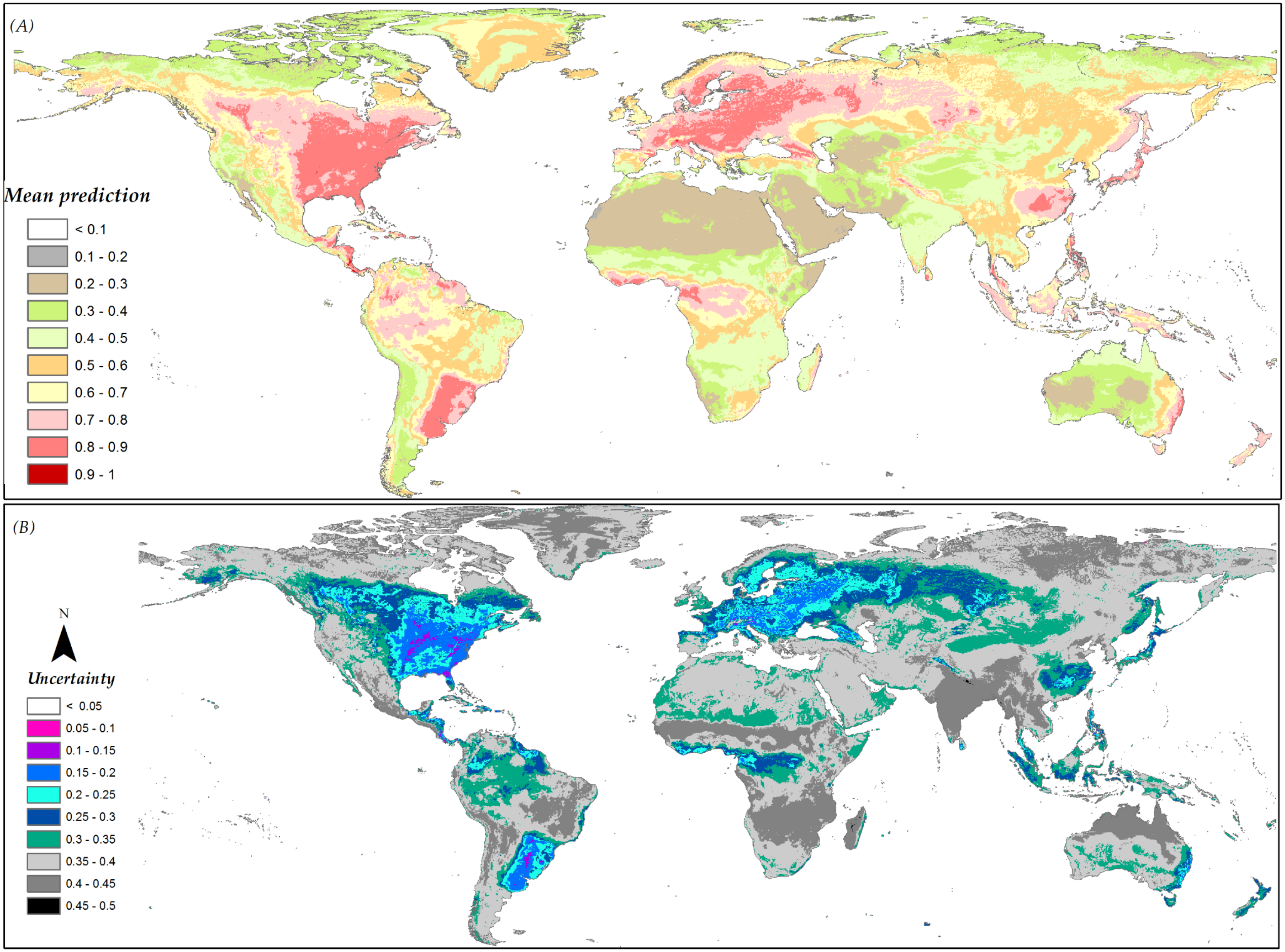

Figure S7.2: Global average prediction (A) and uncertainty map (B) for *D. v. virgifera*

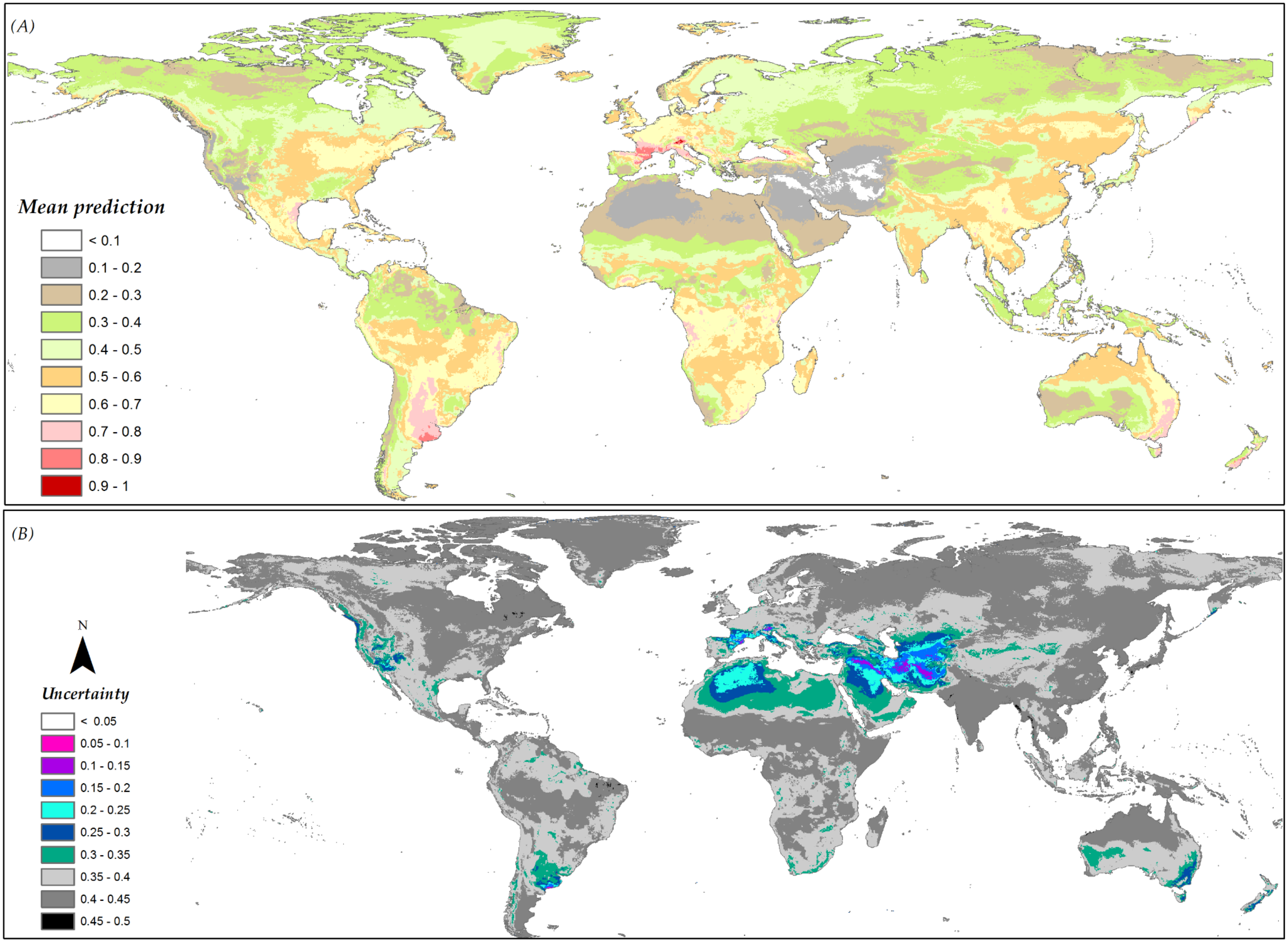

Figure S7.3: Global average prediction (A) and uncertainty map (B) for *T. pityocampa*

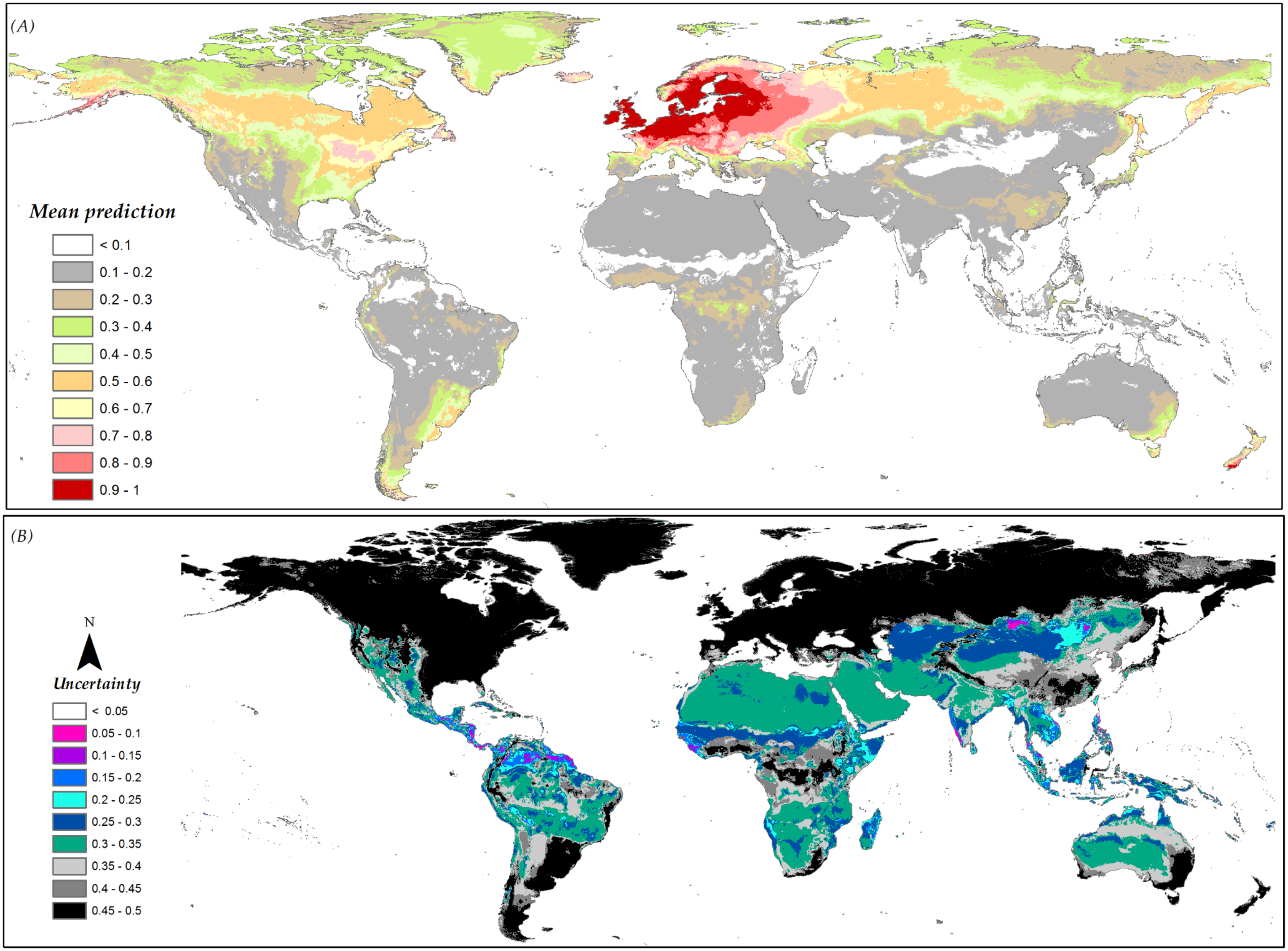

Figure S7.4: Global average prediction (A) and uncertainty map (B) for *V. vulgaris*

Figures S8: Map of uncertainty by modelling components

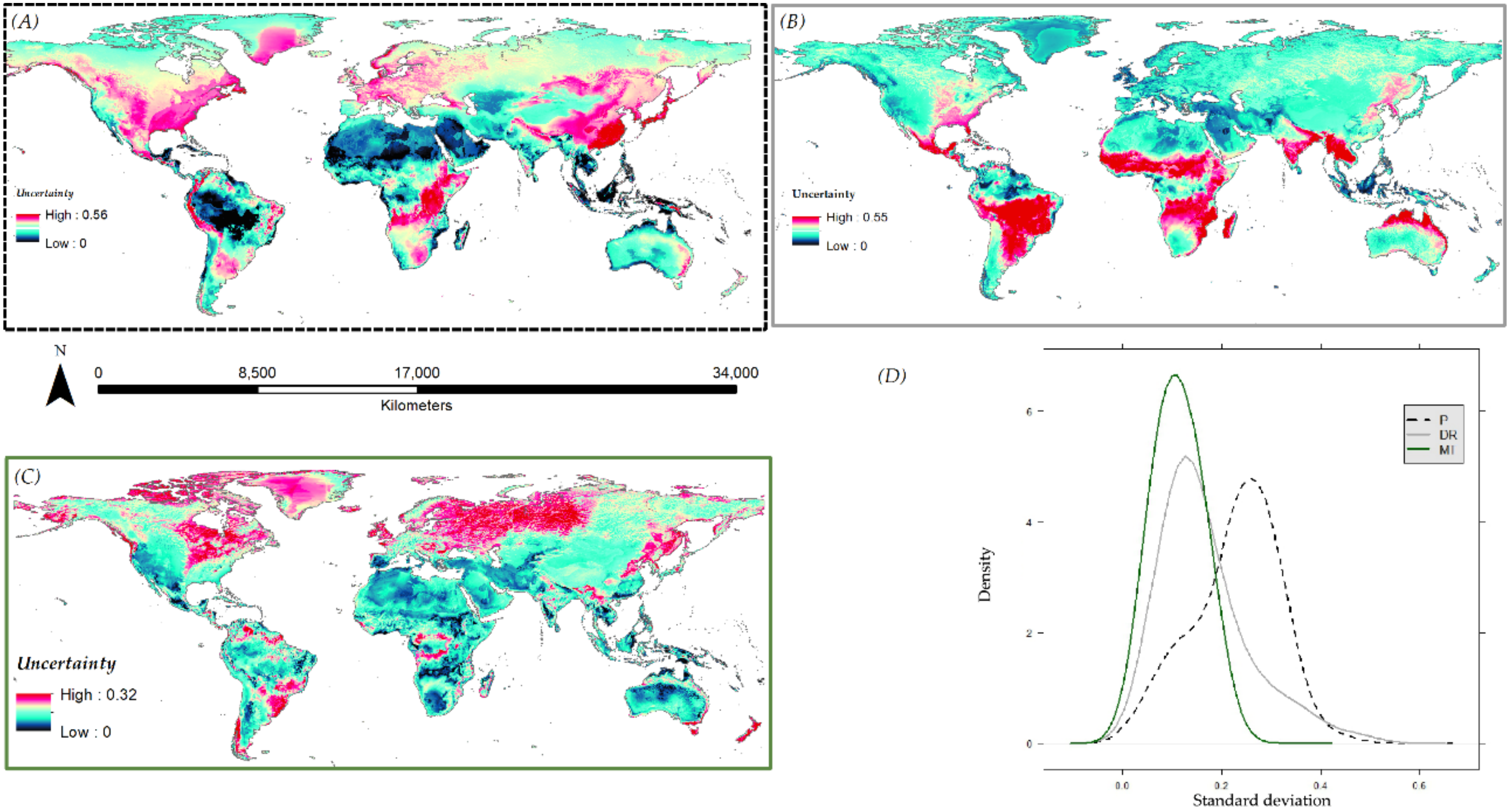

Figure S8.1: Standard deviation of predictions according to predictor data (A) dimension reduction (B) and model type (C) for *A. gracilipes*

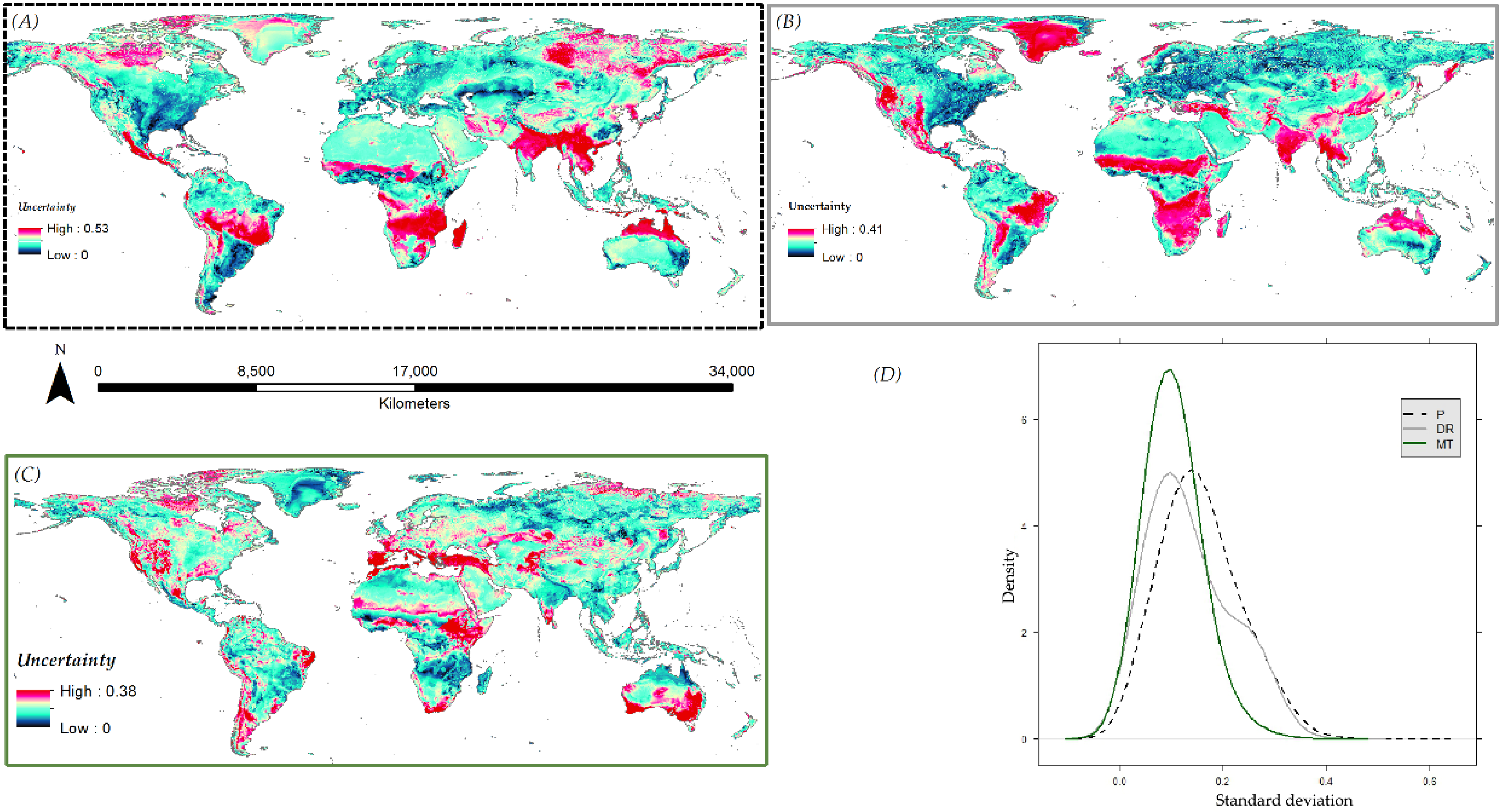

Figure S8.2: Standard deviation of predictions according to predictor data (A) dimension reduction (B) and model type (C) for *D. v. virgifera*

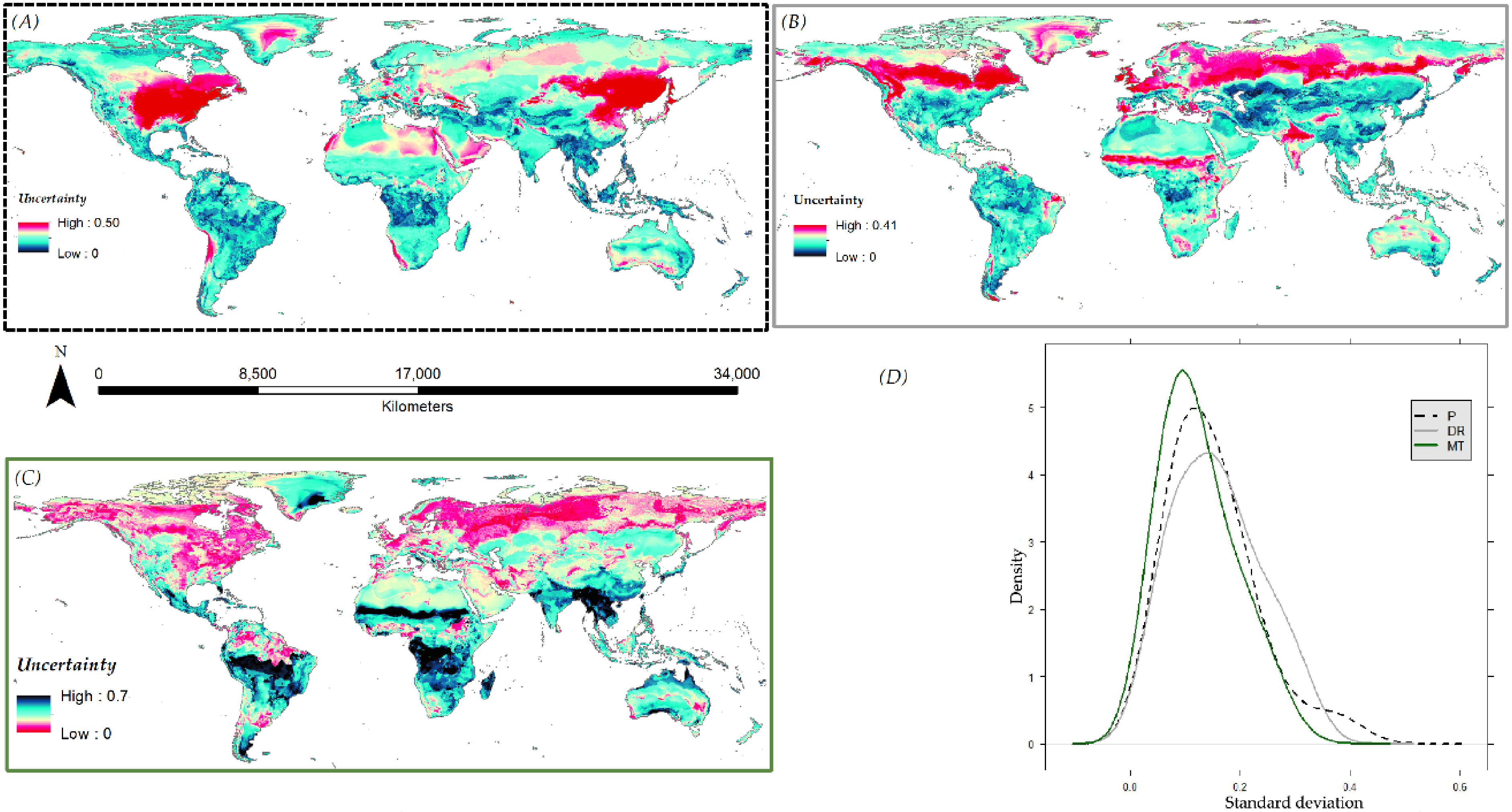

Figure S8.3: Standard deviation of predictions according to predictor data (A) dimension reduction (B) and model type (C) for *T. pityocampa*

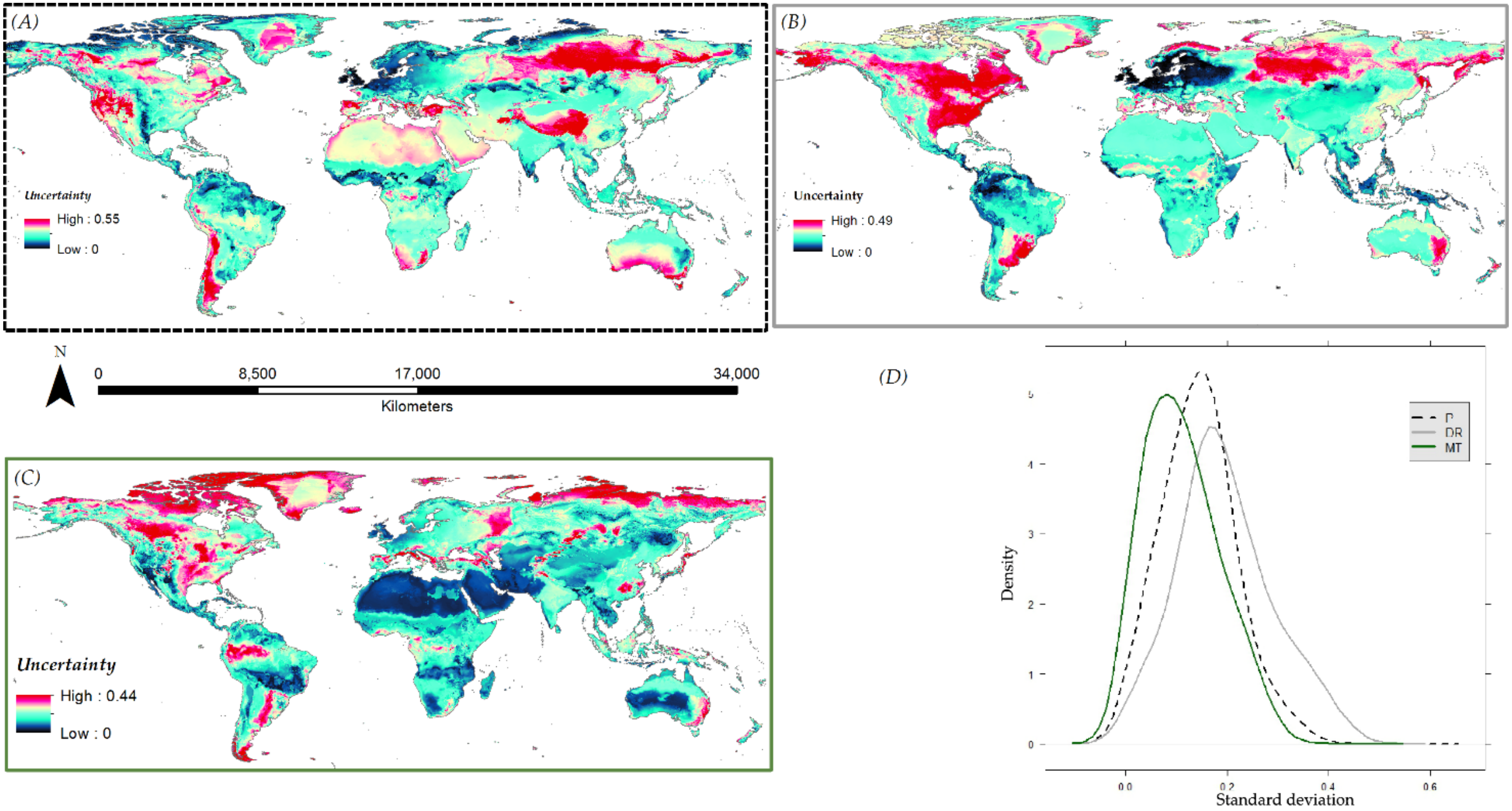

Figure S8.4: Standard deviation of predictions according to predictor data (A) dimension reduction (B) and model type (C) for *V. vulgaris*
